# Engineering Multi-Walled Carbon Nanotube Therapeutic Bionanofluids to Selectively Target Papillary Thyroid Cancer Cells

**DOI:** 10.1371/journal.pone.0149723

**Published:** 2016-02-22

**Authors:** Idit Dotan, Philip J. R. Roche, Miltiadis Paliouras, Elliot J. Mitmaker, Mark A. Trifiro

**Affiliations:** 1 Lady Davis Institute for Medical Research-Jewish General Hospital, Montreal, QC, Canada; 2 Department of Medicine, McGill University, Montreal, QC, Canada; 3 Department of Surgery, McGill University, Montreal, QC, Canada; 4 Division of Endocrinology, Jewish General Hospital, Montreal, QC, Canada; Universidad de Castilla-La Mancha, SPAIN

## Abstract

**Background:**

The incidence of papillary thyroid carcinoma (PTC) has risen steadily over the past few decades as well as the recurrence rates. It has been proposed that targeted ablative physical therapy could be a therapeutic modality in thyroid cancer. Targeted bio-affinity functionalized multi-walled carbon nanotubes (BioNanofluid) act locally, to efficiently convert external light energy to heat thereby specifically killing cancer cells. This may represent a promising new cancer therapeutic modality, advancing beyond conventional laser ablation and other nanoparticle approaches.

**Methods:**

Thyroid Stimulating Hormone Receptor (TSHR) was selected as a target for PTC cells, due to its wide expression. Either TSHR antibodies or Thyrogen or purified TSH (Thyrotropin) were chemically conjugated to our functionalized Bionanofluid. A diode laser system (532 nm) was used to illuminate a PTC cell line for set exposure times. Cell death was assessed using Trypan Blue staining.

**Results:**

TSHR-targeted BioNanofluids were capable of selectively ablating BCPAP, a TSHR-positive PTC cell line, while not TSHR-null NSC-34 cells. We determined that a 2:1 BCPAP cell:α-TSHR-BioNanofluid conjugate ratio and a 30 second laser exposure killed approximately 60% of the BCPAP cells, while 65% and >70% of cells were ablated using Thyrotropin- and Thyrogen-BioNanofluid conjugates, respectively. Furthermore, minimal non-targeted killing was observed using selective controls.

**Conclusion:**

A BioNanofluid platform offering a potential therapeutic path for papillary thyroid cancer has been investigated, with our *in vitro* results suggesting the development of a potent and rapid method of selective cancer cell killing. Therefore, BioNanofluid treatment emphasizes the need for new technology to treat patients with local recurrence and metastatic disease who are currently undergoing either re-operative neck explorations, repeated administration of radioactive iodine and as a last resort external beam radiation or chemotherapy, with fewer side effects and improved quality of life.

## Introduction

During the past decade there has been a significant rise in the incidence of thyroid cancer [1]. This pattern is partly explained by an increase in the detection of small nodules found incidentally on neck imaging, but a more ominous trend is the increased prevalence of larger thyroid (> 4 cm) tumors along with occult lymph node metastases [2]. Papillary thyroid carcinoma (PTC) itself accounts for ~80% of thyroid carcinomas [3, 4]. Despite a very high 10-year survival rate of more than 90% [3], local recurrence occurs in up to 20% of cases, leading to diagnostic and treatment challenges [4]. Additionally, aggressive variants of PTC, such as tall-cell, columnar-cell, insular, trabecular and diffuse sclerosing variants, though rare, are increasing in incidence. These types often require aggressive therapies associated with numerous adverse events [5, 6].

The mainstay of primary PTC treatment is total thyroidectomy [3, 7, 8], usually followed by radioiodine ablation (RAI) in intermediate and high-risk patients [3, 7–10], and lifelong levothyroxine therapy. Although prophylactic central neck lymph node dissection (PCND) remains controversial, therapeutic lymph node dissections are routinely performed [2, 11]. For recurrent/advanced PTC, surgical extirpation is the best option. However, complete biochemical remission with negative thyroglobulin levels is only achieved in 27% of patients (often after multiple interventions) [12], with a 20-year survival rate as low as 36% [13]. The significant number of patients who are not surgical candidates may be subject to adjuvant treatment options, such as external beam radiation therapy (EBRT), that predispose to irreversible morbidities [7, 14–18]. Therefore, it is necessary to find more precise and targeted treatment options that would achieve similar results for primary disease, and improve clinical benefits for recurrent disease, while simultaneously minimizing morbidity.

Unfortunately there are inherent limitations with our current armamentarium of strategies to eradicate tumor recurrence and there is a need to discover new techniques when it comes to recurrent disease. Nanomedicine refers to the use of nanotechnology in the health care domain, and it typically uses materials developed in nanoscale dimensions and already has proven to be extremely effective as a platform for delivery of either physical energy or drugs, and also in imaging applications [19]. Therefore, the notion of nanoparticle-based cancer therapeutics is to circumvent issues with conventional drug pharmacokinetics and resistance while limiting damage, systemically or to normal adjacent tissue. It also extends to include patients who are inoperable based on conventional methods. Based on current chemotherapeutics, increased selective pressure through the application of chemotherapeutic agents leads to increases in tumor resistance [20–22]. In addition, conventional physical therapies used to ablate tissue, such as radiation or high intensity laser treatments, also damage healthy tissue. Nanoparticles are being used as physical agents that are capable of amplifying or converting input energy, to induce cellular damage on a selective scale. This is to their unique photonic properties and plasmonic behavior, most notably carbon nanotube, where such particles absorb light very efficiently and through plasmonic resonance convert its energy absorption to excessive generation of heat at it surface [23, 24].

Bio-affinity nanoparticles, herein described as BioNanofluid, should be able to: 1) efficiently convert light to heat energy, 2) be easily modified with ligands and/or biomolecules to confer specificity, 3) prevent non-specific cell death, and 4) have a size distribution below 1 micron to enable tissue perfusion. The nanomaterial that best fits this description is multiwall carbon nanotubes (MWCNTs), that are cylindrical structures of concentric [25, 26] graphene sheets. The layering of the graphene tube length and large aspect ratio gives a significant surface area for multiple biomolecular attachments, creating multi-dentate particles, where antibodies or other ligands can recognize multiple cell surface receptors. Multiwall carbon nanotubes offer excellent localized temperature gains by virtue of their high capability to absorb light and convert it to heat, while remaining undamaged [27–29]. Heat generated over nanometer scales by nanomaterials affixed to cells, will cause sufficient local hyperthermia without bulk heating of non-cancerous tissue [30]. Furthermore, given that the human body is transparent to near infrared (NIR), such particles with a targeting arm, can deliver an enormous amount of heat locally when exposed to NIR light. NIR already possesses excellent human depth penetration, but can be further extended by fiber/endoscopic advances made in the medical imaging field which can bring NIR light source nearly anywhere in the body [31].

The aim of this study is to design and prepare conjugated BioNanofluids to ablate PTC *in vitro*, by creating a targeted approach with the intent of causing physical damage to cancer cells at the cellular level. In addition, efficacy of a novel targeted photo-thermal therapy for PTC using these conjugated functionalized multi-walled carbon nanotubes (BioNanofluid) in a thyroid cancer cell line model will be assessed.

## Materials and Methods

### Cell lines

The papillary thyroid carcinoma cell line (BCPAP) [32–34] was purchased from DMSZ (Braunschweig, Germany). The hybrid mouse neuroblastoma-motor neuron NSC-34 [35] cell line was donated by Dr. Neil R. Cashman.

### Cell culture

BCPAP cells were cultured in RPMI 1640 media supplemented with 10% FBS. NSC-34 cells were cultured in DMEM media supplemented with 10% FBS and 20% L-glutamine. All cell lines were incubated at 37°C, 5% CO_2_ humidified air in plastic culture flasks (VWR, Canada). Once confluent, cells were collected by using Versene solution (0.48 mM EDTA in PBS), spun down and diluted in media to a concentration of 2.5 to 3.5 x 10^5^ cells/ml.

### Antibodies and chemical reagents

Anti-TSHR antibodies were purchased from Novus Biologicals, Canada, and Acris antibodies Inc, USA. Thyrotropin (Purified human TSH phTSH) was purchased from Bioworld, USA. Thyrogen (Recombinant TSH or rhTSH) was purchased from Genzyme Canada Inc, Canada. Thiolyated PEG 5000 (polyethylene glycol, MW 5000 kD) was purchased from Laysan Bio, USA. NHS (*N*-hydroxysuccinimide) and EDC (ethyl-dimethylaminopropyl-carbodiimide) were purchased from Sigma-Aldrich, USA.

### Western blotting

BCPAP and NSC-34 cells were collected using 0.05% trypsin and lysed using 1 X Reporter Lysis buffer. For Western blot analysis, 20 μg of total-cleared cell lysate was loaded on a 10% SDS-PAGE gel. Primary antibodies were diluted 1:1000 and used under manufacturer’s suggested protocols. The protein expression was visualized using ECL kit and exposed to film.

### Conjugated BioNanofluid preparation

**Fig 1:**
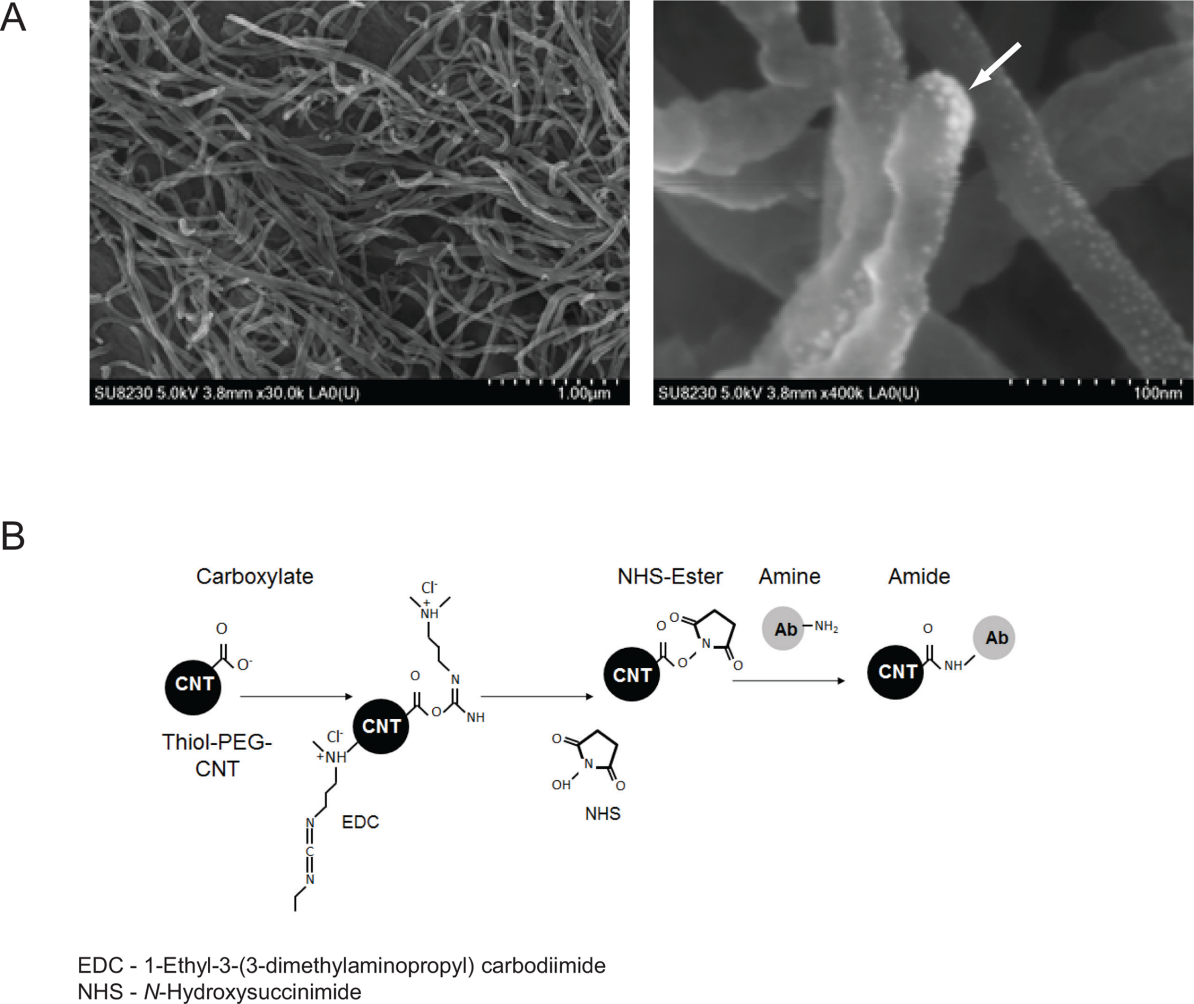
Structure and coupling chemistry of Au-modified MWCNTs. **A.** Scanning electron microscopy (SEM) images of COOH-functionalized Au-labeled Thiol-Carbon derived bionanofluids, at two different magnifications. Au particles have defined spherical structures, highlighted by the arrowhead. **B.** EDC-NHS coupling chemistry to attach bio-affinity molecules, whether antibody or protein/mitogen to the Thiol-PEG-CNT. PEGylation of the Thiol-CNT is described in the Materials and Methods.

COOH-functionalized Au-decorated MWCNTs were obtained from McGill University, Canada, and diluted with d_2_H_2_O to a working concentration of 18–20 mg/L prior to conjugation, to ensure mono-dispersity. COOH functionalization was achieved by plasma treatment, a general method for the addition of functional groups at defects in graphene structures [36, 37]. Au decoration was carried out by pulsed laser ablation using a Nd:YAG laser focused on the MWCNT target at a fluence on the order of 1 J/cm^2^. The process can routinely decorate CdSe, Au, Ag, Si, and Sn on the MWCNTs. The material created had Au-coated islands (variable size 1 nm-5 nm as observed and measured from SEM in **Fig 1A**) for the PEG attachment and exposed COOH groups where Au was absent. Consistency of batch solutions was assessed by UV-vis spectrometry, using the 260 nm peak to determine consistent concentration.

BioNanofluid was modified using Thiolated PEG (MW 5000) over 1 hour with the -SH group forming the Au-S bond, forming the basis for non-specific absorption prevention. The material created had both PEG brushes surrounding the thin gold-coated islands and exposed COOH groups where gold was absent. A stock solution of 150 μM thiolyated PEG was prepared in distilled water at pH 4.5. MWCNT [500 μL stock solution (1 μg)] was incubated with 200 μL of Thiolated PEG5000 150 μM stock solution in a final volume of 700 μL at room temperature for one hour at pH 5. The mixture was then centrifuged for 10 minutes at 13,000 RPM, the supernatant was discarded and the pellet was re-suspended in 350 μL PBS (pH 7.4). The Thiol-PEG-CNT was subsequently conjugated to the targeting molecule. The conjugation mixture included 350 μL of PEG-BioNanofluid, 90 μL (36.8 mM) NHS, 90 μL (22.1 mM) EDC, and 4 μg of one of the following ligands: α-TSHR, Thyrotropin or Thyrogen, with a final pH of 5.5 (see **Fig 1B**). The conjugation was allowed to proceed for 1 hr at room temperature. After completing the conjugation, the mixture was centrifuged for 10 minutes at 13,000 RPM at room temperature, the supernatant removed and the conjugated BioNanofluid pellets were washed (3 times) with PBS and then resuspended in 300 μL PBS.

### Cell targeting and laser treatment

100 to 200 μL of freshly collected cells (containing 250,000–350,000 cells per mL) were mixed with 100–200 μL conjugates, Thiol-PEG-CNT/BioNanofluid or PBS, according to the experiment, in a 1.5 mL Eppendorf tube. The samples were then incubated at 37°C on a rotating rack for 1 hour. The specimens were then washed 3 times with PBS to remove unbound BioNanofluid and extraneous cell debris. After washing, the cells were divided into 25 μL aliquots in 200 μL sterile eppendorfs, and treated with a 532 nm 2.7 W/cm^2^ power laser. Laser treatments were performed using individual incubations of α-TSHR, Thyrotropin or Thyrogen conjugated-BioNanofluid. Experiments were repeated with a minimum of 3 replicates per concentration or laser exposure time. Control experiments were performed with bare IgG-conjugated BioNanofluid, Thiol-PEG-CNT (no ligands) or with PBS and cells alone. The purpose of the controls was to investigate the effects of each chemical and biological modifications, non-specific absorption and exposure time to the laser on cells, and also to limit or eliminate secondary effects that could cause cell death such that it only occurs when the laser interacts with the carbon nanotubes.

Immediately after laser exposure, Trypan blue was added in a 1:1 volume ratio cell fracture in each microtube, and the white (live) cells were counted using a hemocytometer. Counts were performed in triplicate, and each experiment was performed on 3 different occasions. The percentage of cell killing (live cells remaining) was calculated according to equation:

%Live cells=100−(number of live cells post laser treatment/number of live cells pre laser treatment)X100.

### BioNanofluid stability experiments

#### 4°C

Conjugates were prepared on day 1, and kept at 4°C until day 21. Experiments were performed for an entire week (days 1, 2, 3, 4, 5, 6, and 7), and then continued on Days 10, 14 and 21. BCPAP cells were exposed to the 532 nm laser for 30 seconds at a 2:1 cells:BioNanofluid ratio. The concentrations of the conjugated and unconjugated BioNanofluid were measured using the UV-VIS spectrometer, to ensure equivalency of concentration by using a solution of equal absorbance.

#### -20/-80°C

Conjugates created on day 1 were aliquoted and kept at -20°C or -80°C. Experiments were performed on days 1, 5, 7 and then every week for up to 6 weeks. BCPAP cells were exposed to the 532 nm laser for 30 seconds at a 2:1 cell:conjugate ratio. Similarly, the concentrations of the conjugated and unconjugated BioNanofluid were measured using the UV-VIS spectrometer.

## Results

### BioNanofluid characteristics

The optical physics of carbon nanotubes have been studied and described elsewhere [38]. In short, they have the largest absorbance co-efficient of nanoparticle species and a broadband absorbance that fits the design rules. The layering of the graphene tube length is in the micrometer range, conferring a very large aspect ratio for multiple biomolecular attachments, creating multi-dentate particles, where multiple cell surface receptors can be recognized by antibodies or other ligands. Multiwall carbon nanotubes (MWCNTs) offer an excellent method of highly localized heat generation by virtue of their high capability to absorb light and convert it to heat, while remaining undamaged [27–29]. This is a property of graphene-based materials, as they exhibit broadband absorbance of light, being able to absorb a very large spectrum of light colors and being able to convert this energy with high efficiency. This is demonstrated by the black color that the carbon nanotubes exhibit. With absorbance of light, the energy of the photon promotes an electron to a higher energy level from the ground state, the loss of that energy can occur either in weak photo-luminescent emissions and small intersystem processes but the fundamental transfer of energy to the surrounding carbon materials is in the form of heat energy. Thus, heat generated over nanometer scales by bio-functionalized MWCNTs affixed to selective/targeted cells, will cause rapid and sufficient local hyperthermia without “bulk heating” of nearby potentially sensitive tissue. Previously, gold nanoparticles with their plasmonic absorption properties have been used to convert light to heat and induce cell death in tumors [25]. The large light fluxes or ultra-short light pulse modulations required to achieve high temperatures [39] need prolonged exposure of 5 to 15 minutes [40] and cause detrimental damage to surrounding non-cancerous cells.

The base material of the BioNanofluid is COOH functionalized multi-walled carbon nanotubes (length ranging from 0.25 μm to 10 μm with a diameter of 25–50 nm) with gold (Au) being sparsely coated as an additional modification, was generated via amide & thiol linkages for chemical and biological ligands. Images of COOH-functionalized Au-decorated MWCNTs were performed using scanning electron microscopy (**Fig 1A**).

### TSHR targeting of BioNanofluid for PTC cell lines

Thyroid stimulating hormone receptor (TSHR) was chosen for its robust expression in both normal as well as in differentiated thyroid cancer cells [41–44] as evidenced by studies showing no down-regulation of TSH-R in differentiated thyroid cancer cells [45], while others showed TSHR being over-expressed in thyroid carcinomas and benign adenomas as compared to normal thyroid tissue [43]. The attachment of TSH to its receptor stimulates cell growth and proliferation, thereby aiding in PTC progression [46]. This provides an explanation as to why levothyroxine is prescribed in doses that suppress TSH, that is, in order to prevent the growth of micro-metastases and/or remnant thyroid tissue following conventional therapy for thyroid cancer. Furthermore, the robust expression of the TSHR, being ubiquitous to the thyrocyte, still serves as an important and persistent regulator and physiological marker in primary and metastatic disease with the ability to target the BioNanofluid conjugates for a therapeutic potential.

**Fig 2:**
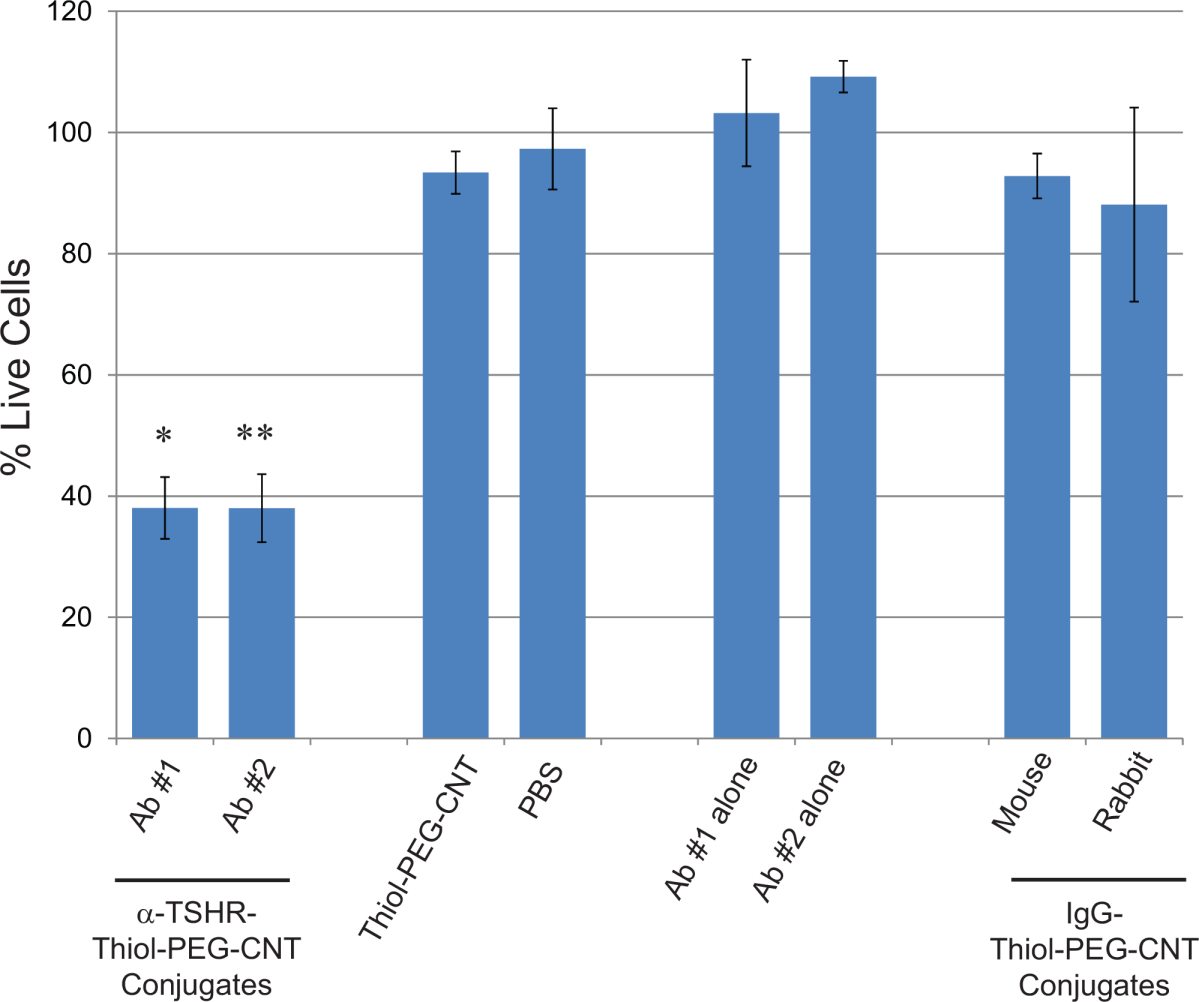
α-TSHR-bionanofluid targeting of BCPAP Papillary Thyroid Cancer Cells. Two TSHR specific antibodies, purchased from different supplies (Ab #1, Acris Antibodies; Ab #2, Novus Biologicals) were conjugated to our Thiol-PEG-CNTs, along with IgG rabbit and mouse-Thiol-PEG-CNT conjugates as non-specific controls for targeting cell killing of BCPAP PTC cells. Other control conditions include PBS, CNT particles and antibodies alone. Results are shown as % Live Cells, following laser treatment followed by Trypan Blue staining to define dead from live cells. α-TSHR-Bionanofluid conjugates significantly (Ab#1, p = 0.000148; Ab#2, p = 5.74 x10^-5^) killed BCPAP cells vs. IgG-bionanofluid conjugates. All other controls showed no significant cells killing rates vs. IgG-bionanofluids.

To assess the potential of targeting the TSHR, a TSHR-positive expressing PTC cell line (BCPAP) was found and incubated with two different α-TSHR-BioNanofluid using TSHR antibodies from different suppliers (**Fig 2**). Both supplier antibodies showed similar and significant cell killing rates of 62±5.6% (Ab#1 –Acris antibodies) and 62±5.1% (Ab#2 –Novus biologicals), with p-values of 0.000148 (Ab#1); and 5.74 x10^-5^ (Ab#2), when compared to IgG-BioNanofluids. The antibodies alone, IgG-BioNanofluid, or unconjugated Thiol-PEG-CNTs showed minimal cell killing potential.

### BioNanofluid optimizations

In order to reach a maximum cell killing rate with a minimum occurrence of non-specific cell death, we proceeded to optimize our conditions to account for cell concentration to BioNanofluid and length of time of exposure of the bionanofluid-cell complex to the laser.

**Fig 3:**
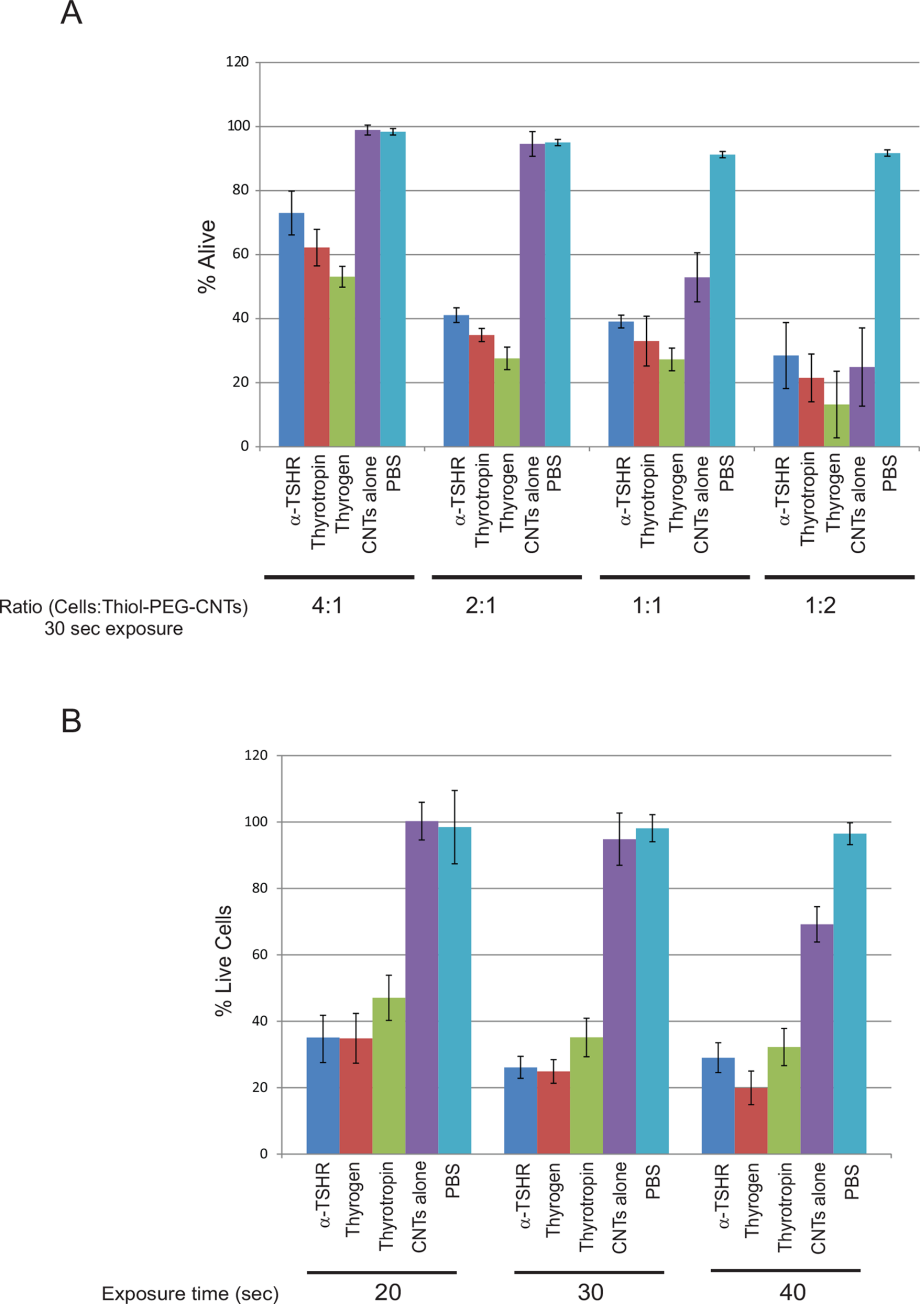
Concentration and time optimization of TSHR targeting bionanofluid to BCPAP cells. Included in these experimental conditions are, α-THSR-, Thyrogen-, and purified Thyrotropin-Thiol-PEG-CNT conjugates. Control conditions included PBS and CNT alone. **A.** Determination of optimal cell to conjugate BioNanofluid ratio to achieve specific maximum targeted BCPAP cell killing. Laser exposure time was 30 seconds for all conditions. Ratios are represented as volume:volume ratios, thus for a 1:1 ratio, 100 μL of cells (of 250,000–350,000 cells per ml) were mixed with 100 μL of Conjugated-BioNanofluid of 2 μg/mL concentration. **B.** Optimal exposure time determination experiment. BCPAP cells were exposed to laser treatment for 20, 30, and 40 seconds, at a 2:1 cell:conjugated- or unconjugated-Thiol-PEG CNT ratio.

First, we evaluated four different ratios (4:1, 2:1, 1:1 and 1:2) of cells to BioNanofluid conjugates. A 2:1 cell to BioNanofluid ratio yielded 58.9% (±2.3) cell killing rate by α-TSHR-BioNanofluid, 65.1% (±2.1) for Thyrotropin-BioNanofluid, and 72.4% (±3.52) for Thyrogen-BioNanofluid (**Fig 3A**). Furthermore, the Thyrogen-BioNanofluid outperformed both α-TSHR- and Thyrotropin-BioNanofluid at the 2:1 ratio. Increasing BioNanofluid content to a 1:1 or 1:2 (cell:BioNanofluid) ratio caused 47.1% (±7.65) and 69.0% (±4.52) cell death, respectively, of non-targeted cell killing rates in the CNT nanoparticles alone control group. This increase of cell death, at higher concentrations of unconjugated nanoparticles relates to an increase in particle retention on the cell by non-specific cell surface associations that are prevalent at all MWCNT concentrations. Therefore, a higher concentration/ratio of the non-specific retention particle group is numerous enough to generate unwanted bulk-heating effects thereby killing cells. Furthermore, the relative additional cell death of non-specific targeting of the α-TSHR-BioNanofluid conjugates at the 1:1 [56.2% (±8.70), p = 0.1501] or 1:2 [61.8 (±21.2), p = 0.681] ratio group was not significant.

Exposure time experiments were performed with all conjugates to determine the highest rate of cell killing without loss of specificity, i.e., high rates of non-targeted cell death (**Fig 3B**). Forty second exposure yielded 67.8% (±4.4) cell killing (α-TSHR), 67.8% (±5.6) (Thyrotropin), and 80.1% (±5.1) (Thyrogen). Thirty second exposure yielded 59.4% (±1.3) killing (α-TSHR), 64.9% (±5.8) (Thyrotropin), and 75.2% (±3.5) (Thyrogen). Twenty second exposure yielded 48.5% (±4.75) (α-TSHR), 52.9% (±6.8) (Thyrotropin), and 65.8% (±7.5) (Thyrogen). Although the highest cell killing rates were achieved with longer exposure times (40 sec > 30 sec > 20 sec), non-targeted killing in the BioNanofluid control group with 40 second exposure time was 32.7% (±11.6), again reflecting the issue of dealing with non-specific cell killing through re-investigation of the PEG-modification. Therefore, a 30 second exposure time corresponded to the highest rate of specific cell killing. However, when laser exposure was increased by a ten second interval, it caused approximately 2.5 times the amount (12% cell death at 30 seconds vs. 33% cell death at 40 seconds of laser exposure) of non-specific cell death in the un-conjugated CNT control group. This reflects the turning point between nano-scale temperature delivery and the time it takes for BioNanofluid to sufficiently bulk heat a cell suspension. Previous studies have examined the effect of laser power and exposure time needed to transfer enough energy leading to cell destruction. For example, one study treated Daudi cells with exposure times of 7 minutes and yielded more than 90% cell death [47]. Other studies treated breast cancer, colon cancer, hepatocellular carcinoma and Daudi cell lines for 3 minutes or more [48–50]. Although some of the aforementioned studies used single-walled carbon nanotubes (SWCNT) with defined photothermal characteristics, we found that even with a small increase of 10 seconds, the use of MWCNT demonstrated more collateral damage. Our findings suggest that the technique and particle preparation of the MWCNT used for this experiment show greater efficiency in photo-thermal heat transfer in a cell-specific fashion.

### TSHR-targeted BioNanofluid selectivity and specificity

**Fig 4:**
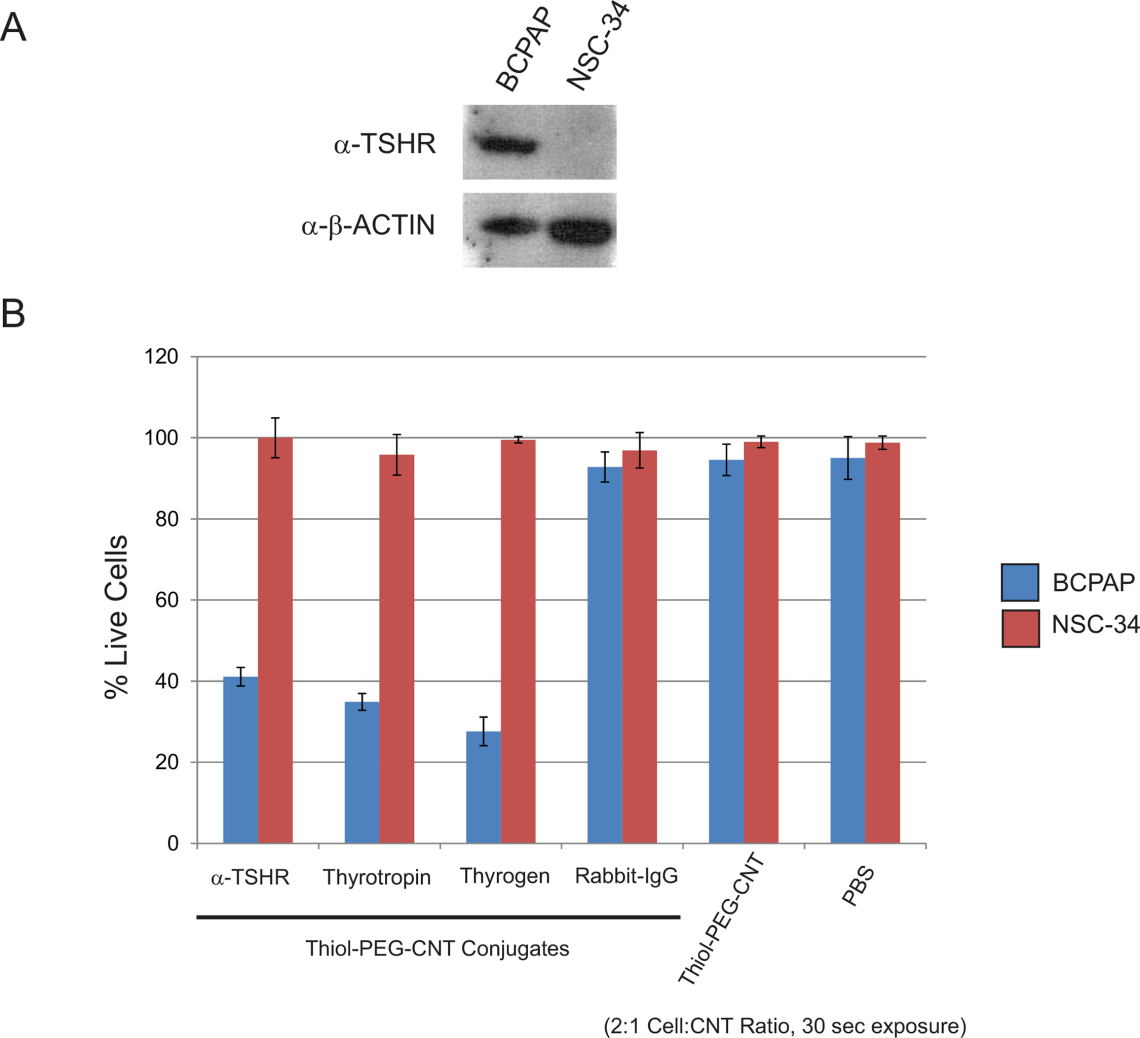
Selective cell killing of BCPAP TSHR-positive vs NSC-34 TSHR-negative cells with TSHR-targeted bionanofluid. **A.** TSHR expression of BCPAP and NSC-34 cells was determined by Western blot analysis, using TSHR specific antibody. BCPAP were positive for TSHR expression, whereas NSC-34 cells were null. B-ACTIN was used as a loading control. **B.** BCPAP and NSC-34 cell were incubated α-THSR-, Thyrogen-, and purified Thyrotropin-Thiol-PEG-CNT conjugates. Control conditions included IgG-thiol-PEG-CNTs, PBS and CNT alone. All conditions were performed in 2:1 cell:bionanofluid ratio and 30 second laser exposure. The BCPAP cells showed ~60% to ~73% cell killing with all TSHR targeted bionanofluid conjugates, whereas minimal cell death was observed with the control other conditions. The NSC-34 cell line showed negligible cell death in all conditions.

To assess both selectivity and specificity of the TSHR-targeted BioNanofluid, we selected to perform the cell ablation experiments simultaneously on both a TSHR positive and TSHR negative cell line. As BCPAP is a positive TSHR expressing cell line, we found that the mouse motor neuron cell line NSC-34 is null for TSHR expression (**Fig 4A**). Therefore, we tested with α-TSHR-, Thyrotropin- and Thyrogen-BioNanofluids against both BCPAP and NSC-34 cells (**Fig 4B**). Using a 2:1 cell:BioNanofluid ratio and 30 seconds, we found that our selective targeting of TSHR can specifically and significantly discriminate between TSHR expressing and non-expressing cell lines.

### BioNanofluid stability

**Fig 5:**
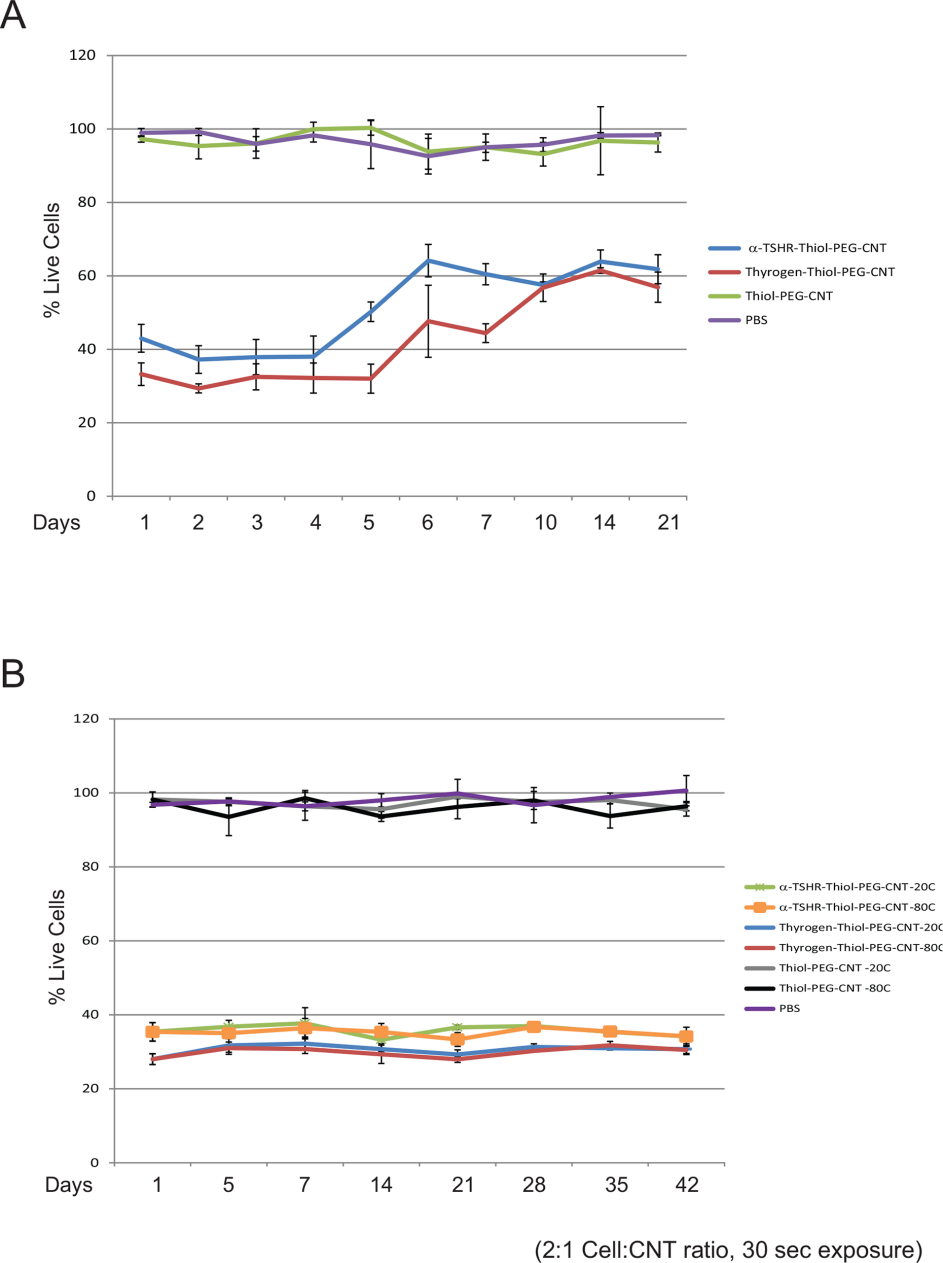
Bionanofluid conjugate stability assessment. **A**. α-TSHR- and Thyrogen-Thiol-PEG-CNT conjugates were prepared on day 1 and kept at 4°C for up to 21 days. Conjugates activity was assessed by cell killing assay of BCPAP cells (as described above). **B.** Similarly, α-TSHR- and Thyrogen-Thiol-PEG-CNT conjugates were prepared on day 1 and were kept at -20°C or -80°C for up to 6 weeks. Conjugates activity was assessed by cell killing assay at day 5, day 7, and every week for up to 6 weeks.

Experiments were performed to evaluate the activity of the TSHR-targeted BioNanofluids, by assessing their stability under prolonged storage conditions. A batch of α-TSHR-BioNanofluid was created and stored at either 4°C for 21 days, or -20°C and -80°C for 6 weeks. The batch of α-TSHR-BioNanofluid stored at 4°C was assessed for their activity to ablate BCPAP cells everyday over a 1-week period, and then repeated on days 10, 14 and 21 (**Fig 5A**). α-TSHR-BioNanofluid began to lose efficacy by day 5, with its ability to ablate BCPAP cells dropping from 60% to 40%, while Thyrogen-BioNanofluid conjugates lost efficacy on day 6. Both α-TSHR- and Thyrogen-BioNanofluid appeared to plateau to being 40% effective until the conclusion of the experiment, suggesting continued but hindered cell selectivity. It can be hypothesized that performance will decline with the denaturation of the protein ligands over a short period of time when stored at unstable conditions of 4°C. However, although nothing can be directly inferred as to Thyrogen or α-TSHR structure, the concentrations of the BioNanofluid stored at 4°C were stable as measured by the UV/VIS spectrometer prior to mixing with cells. As ligand-BioNanofluid UV/VIS absorbance remains constant irrespective of potential denaturation, it is suggested that giving the combined structure’s absorbance at 260 nm (absorbance at this wavelength is common to proteins and CNTs) that little or no loss of ligand or CNTs due to liability of amide attachment or CNT degradation are reasons for the decline in activity (see S1 Table).

A similar experiment was conducted over 6 weeks, with the BioNanofluid stored at -20°C or -80°C (**Fig 5B**). BCPAP cell ablation experiments were performed on days 1, 5, 7 and weekly, for up to 6 weeks. The results reveal stability of the BioNanofluid activity at both -20°C and -80°C storage conditions, as observed by cell ablation percentages for the α-TSHR-BioNanofluid and still maintaining >60% efficacy, and the Thyrogen-BioNanofluid possessing >65% efficacy, over the 6 week course of the experiment. The conjugated and un-conjugated MWCNT concentrations were measured in parallel for each experiment, and all showed stable concentrations.

## Discussion

There are inherent limitations in the treatment of recurrent thyroid cancer. Although the majority of cases are treated with thyroidectomy, followed by TSH-suppressive therapy with levothyroxine and radioactive iodine in selected cases; recurrent thyroid cancer presents a therapeutic challenge. The paradigm of thyroid cancer treatment and recurrence provides an appropriate framework to study the application of molecularly targeted physical agents. Nano-mediated photo thermal therapy is gaining momentum in the form of targeted physical agents that treat a variety of cancers. The critical challenge for thyroid cancer is to deliver an agent that not only targets tumor cells, but also targets normal remnant thyroid cells. In this study, we aimed to assess the efficacy of an innovative targeted physical therapy using newly engineered bio-affinity functionalized carbon nanotubes, or BioNanofluid conjugates, in order to demonstrate *in vitro*, the efficacy of targeting TSHR and ablating a papillary thyroid cancer cell line.

In order to maximize our cell killing rate with a minimum occurrence of non-specific cell death, we have optimized our conditions by determining the cell to BioNanofluid ratio concentrations and time of exposure to activating laser (**Fig 3**). Significant differences in the targeted cell killing rates are evident when comparing between the α-TSHR-, Thyrotropin- and Thyrogen-BioNanofluid conjugates during a 30-second exposure time (59.4% vs. 64.9% vs. 75.2%, p<0.005) (**Fig 4**). Both Thyrotropin and Thyrogen bound with high affinity to the TSHR, as reflected in the percentage of cell death. Our data implies that TSHR antibody may bind with a lower affinity to the TSHR, as less cell killing is observed [51, 52]. The natural TSH, as well as the recombinant one have the same amino acid sequence, attaching to the TSHR within a binding pocket, made from a combination of the α and β subunits [53, 54]. The attachment is thus strong and stable, and less prone to detach. But we conclude further investigation in ligand orientation and steric influences must be considered before a definitive order can be given to ligand binding properties or preference for use with ligand conjugates. The TSHR-targeted BioNanofluid also provides a highly effective and specific cell targeting platform that achieves high cell killing rates in a BCPAP cell line, with negligible cell death in a control TSHR-negative cell line (**Fig 4**). The relevance of limited non-targeted cell death cannot be understated, as even though the approach is to target cancerous and non-cancerous thyroid tissue, other tissue types should not be recognized by the bio-modified nanoparticle agents. However, it is important to note that despite the widespread expression of the TSHR throughout [55–61], the BioNanofluid is activated only when exposed to a given optical power (over wavelengths in the UV-Vis to NIR spectrum) and only to the area exposed in the path of the laser beam. In other words, heat transfer from MWCNT will work more efficiently towards destroying specific cells, with the added benefit of preventing damage to surrounding or distant tissues that may express the same surface markers as they will not be subject to laser exposure of sufficient power to induce thermal effects.

The saturation level of BioNanofluid conjugates to cells has not yet been determined, with respect to how many nanoparticles are required to induce cell death. But the issue of non-specific absorption complicates this analysis as it suggests further work is required to lower the fraction of particles retained by this mechanism. The non-specific absorbance hypothesis has support reflective of incomplete PEGylation of the MWCNT surface caused by an uneven gold distribution on its surface. PEGylation is driven via the Au-S bond formation that can only occur at the gold modification that has proven to be irregularly distributed on MWCNT material. In further work, it will be essential to improve PEGylation as incomplete polymer coating will affect nanoparticle retention and solubility for *in vivo* investigations. In the current study, PEGylation reduces non-specific cell death by approximately 5% as compared to unmodified MWCNT (10% non-specific cell death). This was achieved by a 2:1 cell to conjugate ratio, preventing non-specific cell adhesion by PEGylation of MWCNTs. If the layering is improved in terms of its consistency, it is further possible to consider reducing non-specific attachment and killing at higher MWCNT concentrations, thereby avoiding bulk heating that raises the temperature significantly other than on the cell surface.

In surgical practice the use of high-energy laser sources has been applied to treat cancer by photo-ablation of the tissue in an increasing number of cancer types [62, 63] or used in combination with photodynamic therapy, however both are detrimental to nearby tissue [64]. Several groups have tried to assess the efficiency of physical ablative treatment for cancer using nanoparticles, with different, albeit high, ablative success rates. However, most used prolonged exposure time [47–49, 65–69] and extreme high laser power (up to 64 W/cm^2^) [49, 66, 67], that cannot be applicable *in vivo*. At extreme power levels damage to normal cells in the area of the tumor (even if the particles were targeted successfully to the tumor), may result in substantial collateral damage and possible side effects. Similarly, high particle concentration will create the same effect [30, 50, 65, 70, 71], hence why investigations should focus on driving particle efficiency and lower concentrations thereby allowing much lower laser powers to be used. This has been demonstrated as an efficient method of inducing cell death in this study. Investigations performed on non-ligand and untargeted modified carbon nanotubes has shown that the absorbance of light and the surface temperature of tubes is exceptionally high and with a rapid temperature dispersal in the order of 1 to 2 μm away from the nanoparticles into the cells or tissue. This has been defined and detailed as the “zone of nanoparticle killing” model [72]. Using targeted carbon nantobes the area of tissue damage is extremely localized and nearby damage is of a few cells. Future work will focus on developing ablative treatment from the current large area models applied in radiotherapy and high intensity laser ablation, towards a local scale that moves gradually from millimeter to micron scales. As such, ablative treatments need to start working towards improving clinical applications and away from previous gold nanoparticle formulation studies, where the particle system delivers high specificity, fast action, and efficient heat conversion.

## Supporting Information

S1 Table UV/VIS data of CNT conjugates used cell ablation studies for Fig 5A and 5B. Given is raw UV/VIS spectral data at 260 nm of CNT conjugate preparations used in our stability cell ablation studies. Shown are also graphs representing percent ratio vs. CNT-PEG-EDC UV/VIS concentration for the corresponding day, of each conjugate preparation, over the time course of the assay, with 1.0 OD (260nm) of CNT being approximately 15 ng/μL. For this experiment and all subsequent cell ablation experiments, a minimum of three separate conjugate CNT particles were prepared, and with concentration determined by UV/VIS spectra at 260nm. Similar data was obtained for all experiments illustrated (data not shown). All conjugate particle concentrations were normalized to the CNT PEG-EDC starting material, prior to performing cell ablation experiments.
(XLSX)
