# Correction: Engineering Multi-Walled Carbon Nanotube Therapeutic Bionanofluids to Selectively Target Papillary Thyroid Cancer Cells

**DOI:** 10.1371/journal.pone.0158022

**Published:** 2016-06-16

**Authors:** Idit Dotan, Philip J. R. Roche, Michael Tamilia, Miltiadis Paliouras, Elliot J. Mitmaker, Mark A. Trifiro

Dr. Michael Tamilia is not included in the author byline. He should be listed as the third author and affiliated with Lady Davis Institute for Medical Research-Jewish General Hospital; Montreal, QC, Canada and Division of Endocrinology, Jewish General Hospital; Montreal, QC, Canada. The contributions of this author are as follows: Dr. Tamilia was involved in the very early process of the work describe in the paper, in both discussions and experimental design.

## References

[1] Dotan I, Roche PJR, Paliouras M, Mitmaker EJ, Trifiro MA (2016) Engineering Multi-Walled Carbon Nanotube Therapeutic Bionanofluids to Selectively Target Papillary Thyroid Cancer Cells. PLoS ONE 11(2): e0149723 doi:10.1371/journal.pone.0149723 26901566 10.1371/journal.pone.0149723 PMC4762941

